# Helminth Infection and Commensal Microbiota Drive Early IL-10 Production in the Skin by CD4^+^ T Cells That Are Functionally Suppressive

**DOI:** 10.1371/journal.ppat.1004841

**Published:** 2015-05-14

**Authors:** David E. Sanin, Catriona T. Prendergast, Claire D. Bourke, Adrian P. Mountford

**Affiliations:** Centre for Immunology and Infection, Department of Biology, University of York, York, United Kingdom; National Institute of Allergy and Infectious Diseases, National Institutes of Health, UNITED STATES

## Abstract

The skin provides an important first line of defence and immunological barrier to invasive pathogens, but immune responses must also be regulated to maintain barrier function and ensure tolerance of skin surface commensal organisms. In schistosomiasis-endemic regions, populations can experience repeated percutaneous exposure to schistosome larvae, however little is known about how repeated exposure to pathogens affects immune regulation in the skin. Here, using a murine model of repeated infection with *Schistosoma mansoni* larvae, we show that the skin infection site becomes rich in regulatory IL-10, whilst in its absence, inflammation, neutrophil recruitment, and local lymphocyte proliferation is increased. Whilst CD4^+^ T cells are the primary cellular source of regulatory IL-10, they expressed none of the markers conventionally associated with T regulatory (T_reg_) cells (i.e. FoxP3, Helios, Nrp1, CD223, or CD49b). Nevertheless, these IL-10^+^ CD4^+^ T cells in the skin from repeatedly infected mice are functionally suppressive as they reduced proliferation of responsive CD4^+^ T cells from the skin draining lymph node. Moreover, the skin of infected Rag^-/-^ mice had impaired IL-10 production and increased neutrophil recruitment. Finally, we show that the mechanism behind IL-10 production by CD4^+^ T cells in the skin is due to a combination of an initial (day 1) response specific to skin commensal bacteria, and then over the following days schistosome-specific CD4^+^ T cell responses, which together contribute towards limiting inflammation and tissue damage following schistosome infection. We propose CD4^+^ T cells in the skin that do not express markers of conventional T regulatory cell populations have a significant role in immune regulation after repeated pathogen exposure and speculate that these cells may also help to maintain skin barrier function in the context of repeated percutaneous insult by other skin pathogens.

## Author Summary

The skin is a major barrier protecting the host from pathogen infection, but is also a site for immune regulation. Using a murine model of repeated percutaneous exposure to infectious *Schistosoma mansoni* cercariae, we show that, in the skin, CD4^+^ T cells that do not express markers of conventional regulatory T cells are the main early source of immunoregulatory IL-10 and are functionally suppressive of adaptive immune responses. We demonstrate that the production of regulatory IL-10 in the skin is greatly enhanced after repeated schistosome infection compared to levels present after a single infection and that it limits both neutrophil recruitment and local CD4^+^ T cell proliferation, thereby preventing excessive inflammation and tissue damage. Initially (day 1), IL-10 producing CD4^+^ T cells are reactive towards skin commensal bacteria, although over succeeding days they progressively become specific for schistosome antigens. Consequently, our findings highlight a role for early IL-10 produced by dermal CD4^+^ T cells to mediate immune regulation in advance of later stage chronic infection conventionally associated with the presence of IL-10. Our work provides a mechanistic insight into the triggers of early IL-10 production at barrier sites like the skin, and suggests how tolerance and pathogen clearance might be co-regulated early after exposure to infectious agents.

## Introduction

The skin provides an important first line of defence against infectious pathogens which can gain entry via open wounds/abrasions (e.g. *Staphylococcus aureus* [1]), following injection via insect bites (e.g. *Leishmania sp*. protozoa [2] or filarial nematodes [3]), or via direct percutaneous penetration (e.g. soil transmitted hookworms [4], or the helminth *Schistosoma sp* [5]). As one of the body’s largest tissues, the skin is equipped with a several types of cells with immune function, including myeloid cells [6], epidermal γδ T cells [7] and innate lymphoid cells [8], which alongside other types of cell operate in concert against pathogens, but also provide a mechanism of immune regulation to prevent excessive inflammation and ensure tolerance of commensal microorganisms [9–11]. Regulation of the immune response in the skin is particularly important as this organ is host to at least one billion commensal bacteria per square inch [12,13]. One type of cell in the skin that has been most often associated with immunomodulation are conventional αβ regulatory CD4^+^ T cells, such as Tr1 [14] and FoxP3^+^ cells [15], which are thought to be important following skin exposure, for example, to *Leishmania* [16] and *Plasmodium* [17].

Following percutaneous infection of the skin by schistosome parasites, a balance must be established between providing immune protection for the host whilst preventing excessive tissue damage and promoting wound healing [18–20]. The larval parasite (cercaria) gains entry into the skin aided by the release of excretory/secretory (E/S) products from the pre-and post-acetabular glands [21], which have stimulatory, as well as regulatory effects on cells of the host’s innate immune system [22–24]. Indeed, cercarial E/S antigens specifically promote production of regulatory IL-10 by antigen presenting cells [23,25] (Sanin & Mountford, manuscript in preparation), and by cultures of whole blood cells obtained from infected individuals from endemic areas for schistosomiasis [26].

IL-10 is often linked to the development of immune regulation following chronic infection with both protozoan, as well as helminth parasites [27]. It has a well-characterized role in limiting liver pathology and mediating resistance to the chronic stage of schistosome disease where eggs released by adult worms act as a major stimulus [28–31]. However, the vast majority of experimental studies of schistosome infection focus upon immune events after a single infection despite the knowledge that for many human residents of schistosome-endemic regions, repeated exposure to cercariae is likely to occur during daily domestic contact with contaminated water sources [32]. A recent study using a murine model of repeated exposures to schistosome cercariae prior to the onset of chronic egg deposition, revealed that the skin infection site becomes rich in Th2-associated IL-4, but also immune regulatory IL-10 [18]. Moreover, CD4^+^ T cells in the skin-draining lymph nodes of these repeatedly infected mice became hypo-responsive to schistosome antigens [18], which was alleviated in the absence of IL-10 [33]. These findings suggest that the suppression of early immune responses to cutaneous infection can be elicited by repeated pathogen exposure and could be mediated by IL-10 at the skin site of infection.

In the current study, we show that skin inflammation following repeated schistosome infection is increased in the absence of IL-10, and that the primary cellular source of IL-10 in the skin was from CD3^+^ CD4^+^ T cells. Moreover, IL-10^+^ dermal CD4^+^ T cells from repeatedly exposed mice were functionally suppressive as they were able to reduce the proliferation of responsive skin-draining lymph node (sdLN) CD4^+^ T cells from mice exposed to a single dose of cercariae. Finally, the production of IL-10 in the skin, which derived from a combination of schistosome-specific and commensal microbiota-specific CD4^+^ T cell responses, contributed towards limiting inflammation and tissue damage following infection. Thus, we propose that in the skin, IL-10^+^ CD4^+^ T cells that do not express markers of conventional

T regulatory cells have an important role in immune regulation after repeated percutaneous exposure to schistosome cercariae. This has relevance for a range of other pathogens which infect their hosts through the skin.

## Results

### Absence of IL-10 promotes proliferation of dermal CD4^+^ T cells

**Fig 1:**
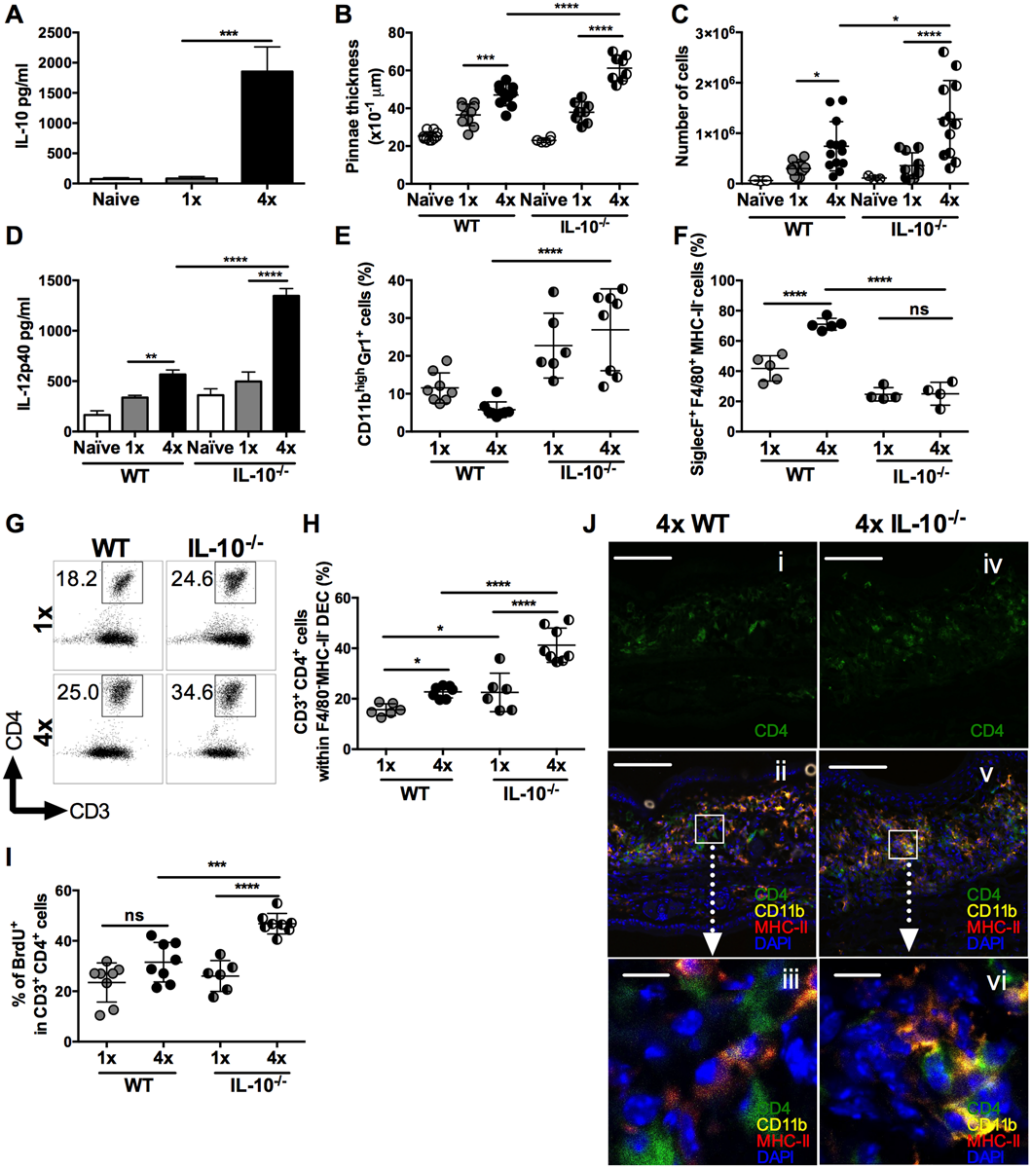
IL-10 produced in the skin following repeated exposures to *S*. *mansoni* cercariae limits CD4^+^ T cell proliferation and mediates recruitment of granulocytes. (**A**) IL-10 released from overnight *in vitro* cultures of skin (pinnae) biopsies from naïve, 1x or 4x infected mice measured by ELISA. Data are means +SEM (6–8 pinnae per group). (**B**) Pinnae thickness (x10^-1^ μm) (n = 8 pinnae) and (**C**) DEC numbers (n = 6–13 pinnae) for naïve, 1x and 4x infected WT, or IL-10^-/-^ mice, on day 4 post-final exposure. Symbols are values for individual tissue samples; horizontal bars are the means ±SEM. (**D**) IL-12p40 detected in overnight cultures of skin biopsies from naive, infected 1x, or 4x WT and IL-10^-/-^ mice. Values are means +SEM (n = 8 pinnae). (**E**) CD11b^high^Gr1^+^ neutrophils as a proportion of DEC recovered from the skin infection site of 1x, or 4x infected WT and IL-10^-/-^ mice. Symbols are values for independent tissue samples; horizontal bars are the means ±SEM; n = 6–8 pinnae. (**F**) Mean percentage ±SEM of SiglecF^+^F4/80^+^MHC-II^-^ eosinophils in DEC recovered from infected 1x, or 4x WT and IL-10^-/-^ mice. Symbols are values for cells obtained from independent tissue samples; horizontal bars are the means ±SEM; n = 4–5 pinnae. (**G**) Representative flow cytometry dot plots and (**H**) mean percentages ± SEM of dermal CD3^+^CD4^+^ T cells (gated on CD11b^-^F4/80^-^MHC-II^-^ DEC) from 1x, or 4x infected WT and IL-10^-/-^ mice on day 4 post-final exposure. (**I**) Percentage of proliferating BrdU^+^ dermal CD3^+^CD4^+^ T cells obtained from 1x, or 4x infected WT and IL-10^-/-^ mice. Symbols are values for cells obtained from independent tissue samples; horizontal bars are the means ± SEM; n = 6–8 pinnae. ANOVA and multiple comparisons tests (Bonferroni’s, Sidak’s and Tukey’s) were performed to compare the means of selected groups (* = p<0.05; ** = p<0.01; *** = p<0.001; **** = p<0.0001; ns = p>0.05). (**J**) Merged confocal Z-stack images (9) from cryosections of pinnae incubated with mAbs specific for CD4 (green), MHC-II (red) and CD11b (yellow), and counterstained with DAPI nuclear stain (blue), from (**i**, **ii & iii**) 4x WT or (**iv, v & vi**) 4x IL-10^-/-^ skin. Scale bar = 100μm (i, ii, iv & v); = 10μm (iii & vi).

Repeated percutaneous exposure to 4 doses (4x) of infective *S*. *mansoni* cercariae causes enhanced production of IL-10 by pinnae skin biopsies compared to those recovered from singly (1x) exposed pinnae (Fig 1A, p<0.001) and was accompanied by increased thickening of the skin at the site of infection when compared to skin exposed to a single dose of cercariae (Fig 1B, p<0.001). However, 4x mice that were deficient for IL-10 (i.e. IL-10^-/-^), had an even greater increase in pinnae thickness compared to 4x wild type (WT) animals (Fig 1B, p<0.0001). This was reflected by the recovery of greater numbers of dermal exudate cells (DEC) from the biopsies of 4x compared to 1x WT skin, which were even more numerous from 4x IL-10^-/-^ compared to 4x WT pinnae (Fig 1C, p<0.05). Skin biopsies from 4x IL-10^-/-^ mice also released significantly more of the pro-inflammatory cytokine IL-12p40 than 1x IL-10^-/-^ and 4x WT skin (Fig 1D, both p<0.0001).

Flow cytometric analysis of myeloid DEC based on F4/80 and MHC-II expression (defined in S1A Fig), combined with mAbs against CD11b and Gr1, or CD4 and CD3 (S1B Fig), revealed marked differences in the leukocyte populations recovered from the skin of WT and IL-10^-/-^ mice (Fig 1E and 1F). The proportion of DEC that were CD11b^high^Gr1^+^ neutrophils increased significantly in 4x IL-10^-/-^ compared to 4x WT skin (Fig 1E, p<0.0001), and when combined with the total numbers of DEC (Fig 1C), the number of neutrophils was 4.74-fold greater in 4x compared to 1x IL-10^-/-^ mice. Conversely, fewer SiglecF^+^F4/80^+^MHC-II^-^ eosinophils were present in DEC from 4x IL-10^-/-^ compared to 4x WT skin samples (Fig 1F and S2A Fig, p<0.0001). There were no differences between the proportions of MHC-II^mid^, or MHC-II^high^ cells with putative antigen presenting function between 4x WT and 4x IL-10^-/-^ skin samples (S2B, S2C and S2D Fig, p>0.05), although there were decreased proportions in 4x compared to 1x skin. Thus, the absence of IL-10 in 4x infected mice resulted in increased inflammation evident as exacerbated thickening of the skin, increased recruitment of neutrophils, and greater levels of IL-12p40.

In addition to changes in innate immune cell populations, the proportion of CD3^+^CD4^+^ (F4/80^-^MHC-II^-^) T lymphocytes in the DEC population was increased in 4x WT compared to 1x WT skin samples (Fig 1G and 1H), and they were much more abundant as a proportion of total DEC recovered from 4x IL-10^-/-^ compared to 4x WT skin (Fig 1H). Furthermore, the proliferation of CD3^+^CD4^+^ DEC, as measured by the *in vivo* incorporation of BrdU, was markedly higher in 4x IL-10^-/-^ pinnae compared to all other groups of infected mice (Fig 1I, p<0.001–0.0001), illustrating that there is greater proliferation of dermal CD4^+^ T cells in IL-10 deficient skin.

The increase in CD4^+^ T cell numbers in 4x infected skin compared to a single exposure quantified by flow cytometry was confirmed qualitatively using immunohistochemistry and confocal microscopy. Pinnae from 4x WT mice had increased numbers of CD4^+^ T cells and CD11b^+^MHC-II^+^ cells compared to 1x WT mice (S3A and S3B Fig). Furthermore, increased numbers of CD4^+^ T cells were apparent in the site of infection of 4x IL-10^-/-^ compared to 4x WT mice (Fig 1Ji *versus* 1Jiv, and S3C Fig). In addition, whilst CD4^+^ T cells in 4x WT skin, were distributed fairly evenly throughout the tissue section (Fig 1Jii and 1Jiii), in 4x IL-10^-/-^ pinnae CD4^+^ T cells were localized in close proximity to CD11b^+^MHC-II^+^ cells (Fig 1Jv and 1Jvi).

### CD4^+^ T cells in the skin are the main source of IL-10

**Fig 2:**
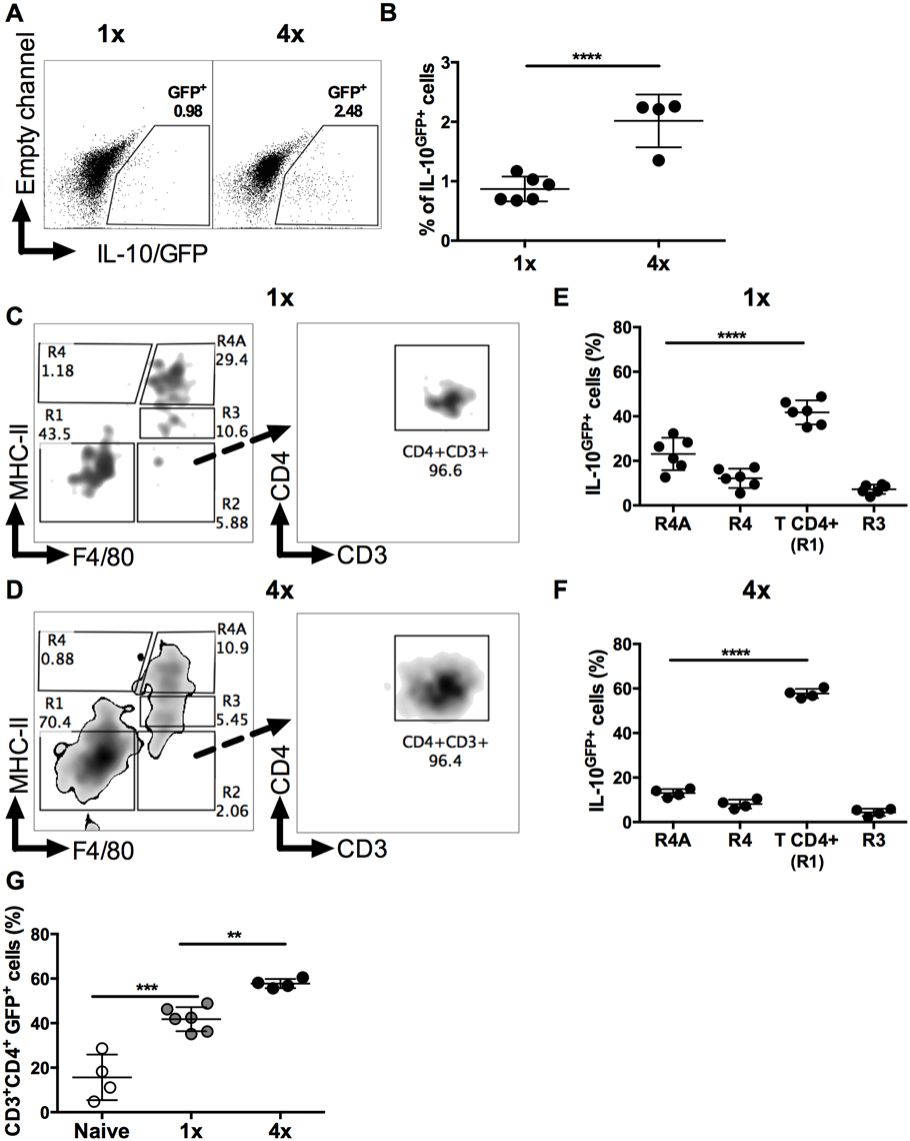
CD4^+^ T cells are the predominant source of IL-10 in DEC. (**A**) Representative flow cytometry dot plots, and (**B**) percentages ± SEM (n = 4–6 pinnae), of IL-10^GFP+^ DEC from 1x and 4x infected mice; gate shows proportions IL-10^GFP+^ cells. Representative flow cytometry dot plots of IL-10^GFP+^ DEC from infected (**C**) 1x and (**D**) 4x IL-10^+/GFP^ mice on day 4 after final exposure to infectious cercariae. Density plots show initial F4/80 *vs*. MHC-II gating strategy (left-hand plots) to yield R1 (double negative F4/80^-^MHC-II^-^), R2 (F4/80^+^MHC-II^-^), R3 (F4/80^+^MHC-II^mid^), R4 (F4/80^-^MHC-II^high^), and R4A (F4/80^+^MHC-II^high^) cell populations. R1 was then gated according to CD4 *vs*. CD3 expression (right-hand plot). Proportions of IL-10^GFP+^ DEC from infected (**E**) 1x and (**F**) 4x mice. (**G**) Proportion of IL-10^GFP+^ CD3^+^CD4^+^ T cells of total IL-10^GFP+^ cells in DEC from naïve, 1x or 4x infected mice on day 4 post-final infection. Symbols are values for cells obtained from independent tissue samples; horizontal bars are the means ± SEM; n = 4–6 pinnae. ANOVA and multiple comparisons tests (Sidak’s and Tukey’s) were performed to compare the means of selected groups (** = p<0.01; *** = p<0.001; **** = p<0.0001; ns = p>0.05).

Using IL-10 reporter mice [34] the proportion of IL-10^GFP+^ DEC recovered from skin exposed to *S*. *mansoni* cercariae was found to be significantly increased in 4x compared to 1x mice (Fig 2A and 2B, p<0.0001). Further characterization of IL-10^GFP+^ DEC (gating strategy in S1 Fig), revealed two IL-10^GFP+^ populations identified as F4/80^+^MHC-II^high^ tissue resident macrophages (R4A gate), and CD3^+^CD4^+^ T cells (from F4/80^-^MHC-II^-^ gate (R1)) in both 1x and 4x pinnae (Fig 2C and 2D). In naïve skin, IL-10^GFP+^ DEC were almost exclusively within the F4/80^+^MHC-II^high^ R4A cell population (S4A Fig). However, one day after infection, the proportion of IL-10 producing cells was equally split between CD4^+^ T cells and F4/80^+^MHC-II^high^ macrophages (S4B Fig). By day 4 after infection, CD4^+^ T cells became the predominant source of IL-10 in both 1x (Fig 2E) and 4x (Fig 2F) infected pinnae. Importantly, the proportion of IL-10^GFP+^ cells that were CD4^+^ T cells was greater in 4x compared to 1x DEC (Fig 2G, p<0.01).

### Conventional CD4^+^ T regulatory cells in the skin are not a major source of IL-10 in 4x infected mice

**Fig 3:**
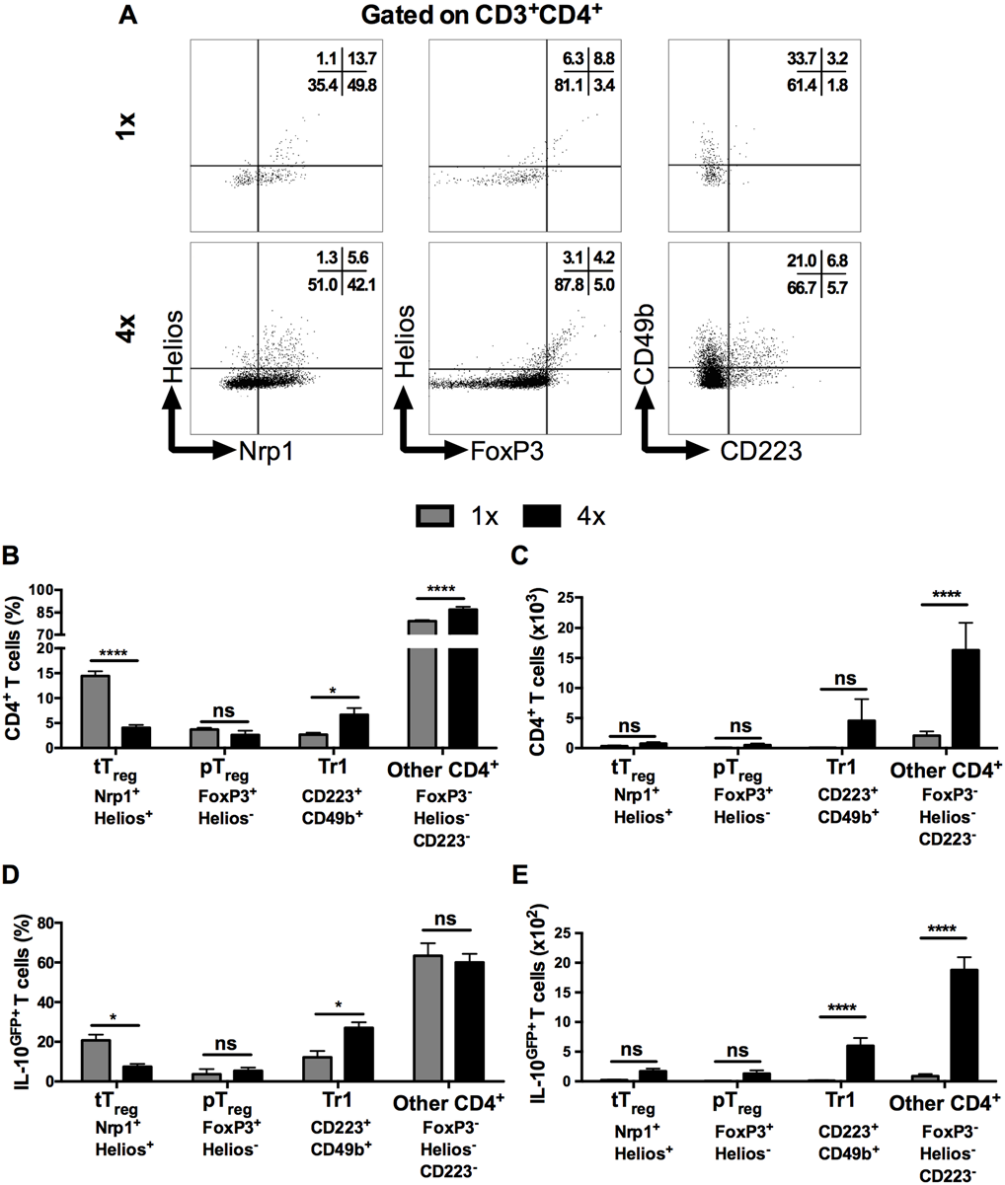
IL-10 producing dermal CD4^+^ cells do not express markers of conventional regulatory T cells. (**A**) Representative flow cytometry dot plots for thymic (Nrp1^+^Helios^+^) T_reg_ cells, peripheral (FoxP3^+^Helios^-^) T_reg_ cells, and Tr1 (CD223^+^CD49b^+^) cells. (**B**) Mean proportion +SEM and (**C**) absolute numbers of dermal CD4^+^ thymic and peripheral T_reg_ cells, as well as Tr1, and other CD3^+^CD4^+^ (Helios^-^FoxP3^-^, not co-expressing CD223 plus CD49b) dermal cell populations recovered from 1x (grey), or 4x (black) infected mice on day 4 post-final exposure. (**D**) Mean proportions +SEM and (**E**) absolute numbers of dermal T cells defined in **A** gated on IL-10^GFP+^ CD4^+^ T cells from 1x or 4x infected mice. ANOVA and Sidak’s multiple comparisons test were performed to compare the means of selected groups (* = p<0.05; **** = p<0.0001; ns = p>0.05).

Several types of CD4^+^ T cells can produce IL-10, including regulatory T cells [35], but the proportion of FoxP3^+^ thymic T regulatory cells (tT_reg_), identified by their co-expression of Helios and Nrp1 in DEC (Fig 3A left panel), was significantly lower in CD3^+^CD4^+^ DEC recovered from 4x compared to 1x mice (Fig 3B, p<0.0001), although the absolute number of tT_reg_ remained unchanged between these two groups of mice (Fig 3C). The proportions and numbers of peripheral T_reg_ (pT_reg_), expressing FoxP3 but not Helios (Fig 3A middle panel), were not significantly different between 1x and 4x DEC (Fig 3B and 3C, p>0.05). Conversely, Type 1 regulatory T (Tr1) cells, which co-express CD223 and CD49b (Fig 3A, right panel), were slightly increased in 4x compared to 1x DEC as a proportion (Fig 3B, p<0.05), but the difference in the total number of Tr1 cells between the infection groups did not reach statistical significance (Fig 3C, p>0.05). Nevertheless, CD4^+^ T cells that did not fall into any of these three categories of conventional regulatory T cells were the most abundant type of CD4^+^ cells in both groups of infected skin (~70% of all CD4^+^ DEC, Fig 3B and 3C), whilst their proportion and number in 4x pinnae was significantly greater than in 1x skin (Fig 3B and 3C, p<0.0001).

Analysis of IL-10^GFP+^ CD4^+^ DEC showed that IL-10 producing pT_reg_ were not a prominent source of IL-10 (<5% of all IL-10^GFP+^ cells) in either 1x or 4x infected skin (Fig 3D). Their number also did not differ significantly between the two groups of mice (Fig 3E). Furthermore, tT_reg_ contributed proportionally less IL-10 relative to other CD4^+^ cell types in 4x skin (Fig 3D), and their number was not significantly different between the two infection groups (Fig 3E). Conversely, the proportion of Tr1 cells in 4x pinnae that were IL-10^GFP+^ increased (Fig 3D, p<0.05), and the difference in their number between 1x and 4x mice was significantly greater (Fig 3E). However, CD4^+^ T cells that fell in none of these categories were by far the predominant CD4^+^ cell population that were IL-10^GFP+^ in the skin after infection, representing more than 60% of all IL-10^GFP+^ CD4^+^ T cells in both 1x and 4x DEC (Fig 3D), as well as increasing in number significantly in 4x DEC (Fig 3E, p<0.0001).

### Dermal CD4^+^ T cells are specific for *S*. *mansoni* antigen and skin commensal microbiota

**Fig 4:**
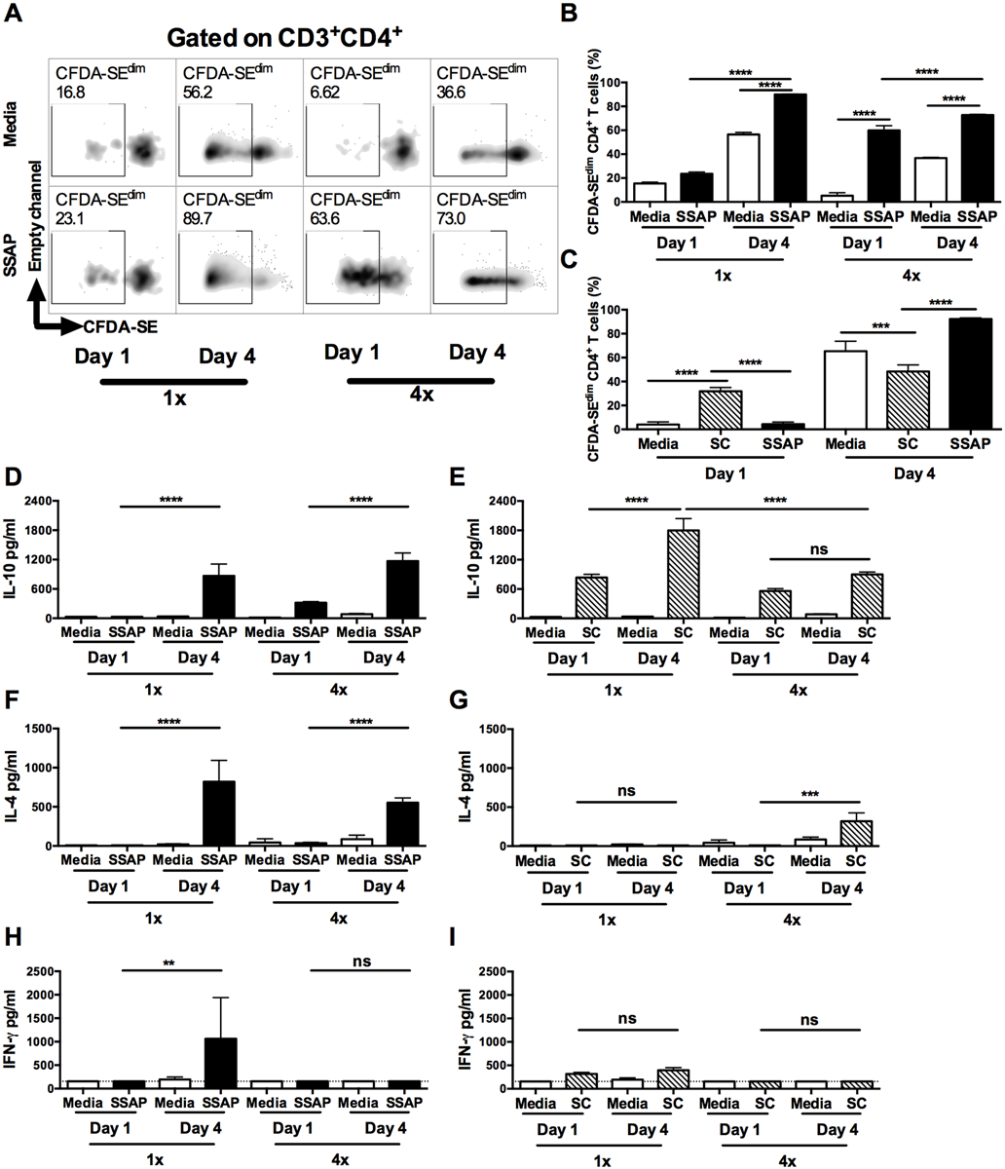
Dermal CD4^+^ T cells from skin exposed to *S*. *mansoni* cercariae respond *in vitro* to parasite and skin commensal antigens. (**A**) Representative flow cytometry density plots of CFDA-SE labelled CD3^+^CD4^+^ dermal T cells from 1x or 4x infected WT mice recovered on day 1 or 4 after infection; DEC were obtained from skin biopsies and stimulated *in vitro* for 96h in the presence, or absence of parasite antigen (SSAP). Gate is set for CFDA-SE^dim^ cells previously gated on CD3^+^CD4^+^cells. (**B**) Percent CFDA-SE^dim^ CD3^+^CD4^+^ cells (mean +SEM; n = 4–5). (**C**) Mean percentage +SEM of CFDA-SE^dim^ CD3^+^CD4^+^ T cells from DEC cultures in the presence or absence of SSAP, or antigen from skin commensals (SC), from 1x infected mice on day 1 or 4 after infection. Production of (**D** & **E**) IL-10, (**F** & **G**) IL-4 and (**H** & **I**) IFN-γ in culture supernatants of skin biopsies from 1x or 4x infected mice obtained on day 1 and 4 post-final infection, cultured in the presence, or absence of SSAP or SC antigen; bars are means + SEM, n = 4–5. ANOVA and Sidak’s multiple comparisons test analysis show statistically significant differences between selected groups (* = p<0.05; ** = p<0.01; *** = p<0.001; **** = p<0.0001; ns = p>0.05).

To verify the antigen specificity of dermal CD4^+^ T cells, carboxyfluorescein diacetate succinimidyl ester (CFDA-SE) labelled DEC cultures were stimulated with soluble antigen derived from parasite larvae (i.e. SSAP). CD4^+^ DEC obtained from 1x mice on day 1 after infection exhibited very low levels of proliferation to parasite antigen, which was similar to those in DEC cultured without antigen (no-antigen controls; Fig 4A left and Fig 4B). In contrast, CD4^+^ DEC from 1x mice recovered on day 4, as well as those from 4x mice recovered on day 1 and day 4, all exhibited enhanced proliferation in response to SSAP (Fig 4A and 4B, p<0.0001). The proliferative responses to SSAP of dermal CD4^+^ T cells recovered on day 4 after infection, from both 1x and 4x mice, were significantly greater than for cells recovered on day 1 (Fig 4B, p<0.0001).

Although dermal CD4^+^ T cells from 1x pinnae recovered on day 1 after infection did not proliferate in response to SSAP, a proportion of them were positive for IL-10 compared to naive mice (S4B and S5A Figs). As it is unlikely that sufficient time will have elapsed for parasite antigen-specific CD4^+^ T cells to have been primed in the lymph node and recruited to the skin site of infection, it was thought possible that these CD4^+^ T cells were responding to other foreign antigens. Commensal microbiota resident on the skin surface might gain access to the dermis as the parasite embarks on percutaneous penetration, thus commensals are a possible source of antigen. Indeed, dermal CD4^+^ T cells from 1x mice recovered on day 1 proliferated *in vitro* to antigen derived from skin commensal microbes (SC), and the level of proliferation was significantly greater compared to that in response to parasite SSAP antigen (Fig 4C, p<0.0001). In contrast, dermal CD4^+^ T cells from 1x mice recovered on day 4 after infection proliferated vigorously to parasite SSAP antigen, to a much greater level than in response to SC (Fig 4C, p<0.0001). CD4^+^ T cell proliferation where no antigen was added is likely to be a result of *in situ* priming of DEC antigen presenting cells with schistosome antigen already present in the skin of repeatedly exposed mice, as larvae can remain in this tissue for up to 4 days post-exposure.

Stimulation of DEC from 1x and 4x infected mice with parasite SSAP antigen resulted in the production of significantly greater quantities of IL-10 compared to unstimulated 1x and 4x DEC, whilst the levels of IL-10 were significantly greater for both groups of mice on day 4 than day 1 (Fig 4D, p<0.0001). High levels of IL-10 were also detected in the supernatants of DEC stimulated with SC antigen, and were at their greatest on day 4 in 1x mice (Fig 4E, p<0.0001). On the other hand, whilst IL-4 was produced by DEC from both 1x and 4x mice in response to SSAP (Fig 4F), detectable levels of IL-4 were only produced by 4x DEC recovered on day 4 in response to SC antigen (Fig 4G, p<0.001). Furthermore, whilst 1x DEC on day 4 produced significant quantities of IFN-γ to SSAP antigen (Fig 4H), only very low levels of this cytokine were detected in DEC supernatants from 1x and 4x mice at both time points in response to SC antigen (Fig 4I).

There were significantly more IL-10^GFP+^ CD4^+^ T cells in DEC recovered on day 1 after *S*. *mansoni* infection than in naïve skin (S5A Fig). However, dermal CD4^+^ T cells in naïve skin might be exposed to commensal microorganisms during the course of the host’s development [36]. Consequently, although very few CD4^+^ T cells were recoverable from naïve skin, we show that proliferation of these cells *in vitro* to SC antigen was comparable to that of dermal CD4^+^ T cells recovered on day 1 after exposure to schistosome cercariae (S5B Fig). Furthermore, cultures of DEC from naive mice stimulated with SC antigen also produced significantly elevated levels of IL-10 (S5C Fig), but did not produce IL-4 (S5D Fig), and only non-significant quantities of IFN-γ (S5E Fig). DEC from naïve mice did not respond to SSAP. Thus, we demonstrate that a number of CD4^+^ T cells with specificity to commensal antigens are present in naïve skin and we conclude that infection with schistosome cercariae enhances the exposure of CD4^+^ T cells to SC antigen resulting in the observed IL-10 production.

### IL-10 production from CD4^+^ T cells is required for regulation of the dermal immune response

**Fig 5:**
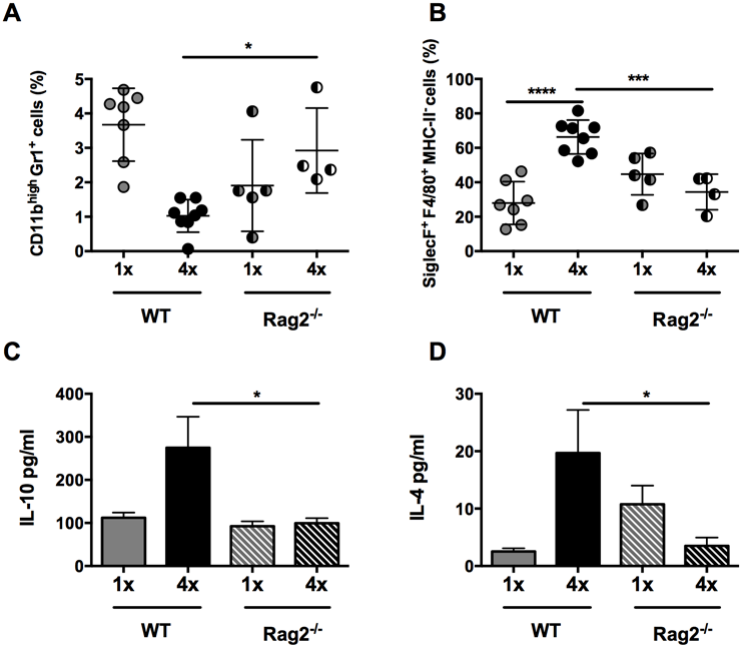
Lack of CD4^+^ T cells ablates IL-10 production induced in skin exposed to *S*. *mansoni* cercariae. (**A**) Proportions of CD11b^high^Gr1^+^ neutrophils and (**B**) SiglecF^+^F4/80^+^MHC-II^-^ eosinophils in total DEC recovered from 1x, or 4x infected WT and Rag2^-/-^ mice on day 4 post infection. Symbols are values for cells obtained from independent tissue samples; horizontal bars are the means ± SEM; n = 4–6 pinnae per group. Production of (**C**) IL-10 and (**D**) IL-4 in overnight culture supernatants of skin biopsies from 1x or 4x infected WT and Rag2^-/-^ mice obtained on day 4 post-final infection; bars are means + SEM, n = 4–6 pinnae per group, as measured by ELISA. Significant differences means were compared between of selected groups by ANOVA and post-hoc tests (Sidak’s and Tukey’s) (* = p<0.05; ** = p<0.01; *** = p<0.001; **** = p<0.0001; ns = p>0.05).

As the production of IL-10 in our model derived predominantly from dermal CD4^+^ T cells, the effect of their absence was examined in repeatedly infected Rag2^-/-^ mice. CD3^-^B220^+^ B cells and CD3^+^CD8^+^ lymphocytes, which are also absent in Rag2^-/-^ mice, were not abundant in *S*. *mansoni* infected skin (S6 Fig). As CD3^+^CD8^+^ lymphocytes were significantly reduced in number in 4x DEC (p<0.001), and CD3^-^B220^+^ B cell numbers remained unchanged, we conclude that differences observed in Rag2^-/-^ mice would be predominantly due to the absence of CD4^+^ T cells. Indeed, the absence of CD4^+^ T cells in 4x Rag2^-/-^ mice resulted in an increase in the proportion of CD11b^+^Gr1^+^ neutrophils compared to 4x WT controls (Fig 5A, p<0.05), whereas there was a significant reduction in the proportion of SiglecF^+^F4/80^+^MHC-II^-^ eosinophils (Fig 5B, p<0.001), reminiscent of the findings for infected IL-10^-/-^ mice (Fig 1E and 1F). The production of IL-10 (Fig 5C, p<0.05) and IL-4 (Fig 5D, p<0.05) by cultured skin biopsies was also impaired in 4x Rag2^-/-^ mice. Collectively, our data are compatible with the notion that the dermal CD4^+^ T cell population is the main source of IL-10 in *S*. *mansoni* infected skin and that other IL-10^+^ myeloid cells (such as those defined in Fig 2) are not sufficient to recapitulate the immune response observed in 4x WT mice.

### IL-10^+^ dermal CD4^+^ T cells suppress proliferation of CD4^+^ T cells in the skin draining lymph node

Since the presence of IL-10 in the skin is able to regulate the proliferation of CD4^+^ T cells *in situ* in the skin site of infection (Fig 1I), we sought to determine whether dermal IL-10 producing cells from 4x mice were able to inhibit *in vitro* proliferation of CD4^+^ T cells recovered from sdLN of mice exposed to 1x infection.

**Fig 6:**
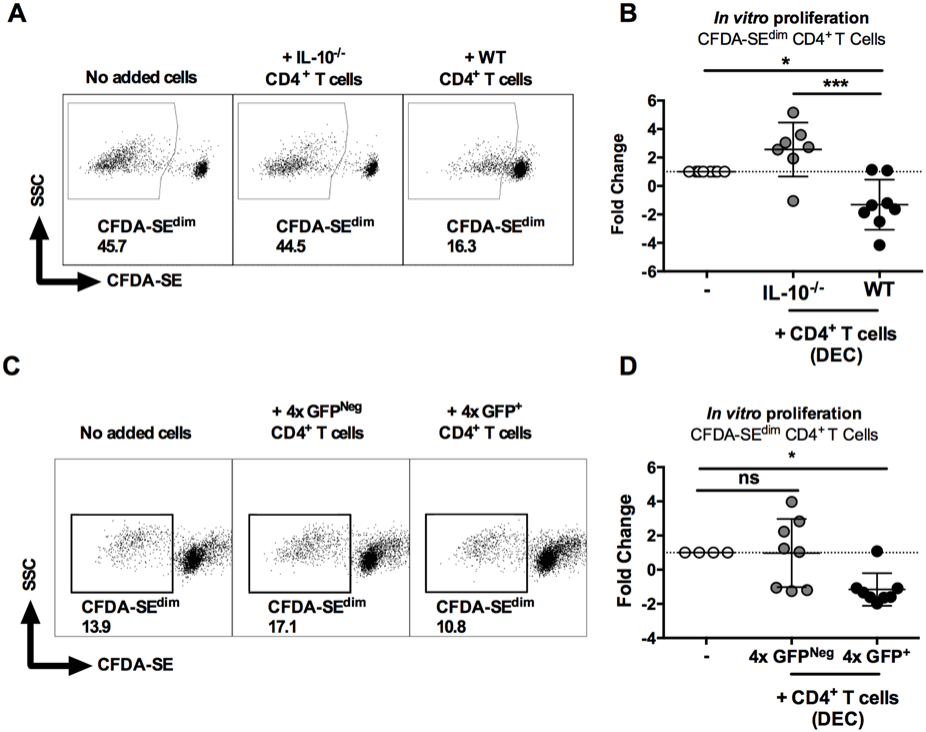
IL-10^GFP+^CD4^+^ T cells from the skin of mice infected with *S*. *mansoni* suppress proliferation of CD4^+^ T cells in the skin draining lymph nodes. (**A**) Representative flow cytometry dot plots of CFDA-SE^dim^ CD3^+^CD4^+^ cells from the sdLN of 1x infected mice after co-culture for 96h with dermal CD4^+^ T cells from 4x infected WT or IL-10^-/-^ mice in the presence of parasite antigen SSAP. (**B**) Data is given as sdLN CD3^+^CD4^+^ T cell proliferation expressed as the fold-change observed in each of seven corresponding mice co-cultured in the presence of dermal CD4^+^ T cells from either WT or IL-10^-/-^ mice relative to sdLN CD3^+^CD4^+^ T cells cultured without added dermal CD4^+^ T cells. Symbols are individual mice; horizontal bars are means ± SEM; n = 7. (**C**) Representative flow plots and (**D**) fold change ± SEM of CFDA-SE^dim^ sdLN CD3^+^CD4^+^ T cells from 1x infected mice (n = 8) co-cultured with GFP^+^, or GFP^Neg^ dermal CD4^+^ T cells from 4x infected mice in the presence of SSAP. Differences between the means of selected groups were compared by ANOVA and Tukey’s multiple comparisons test analysis (* = p<0.05; ** = p<0.01; *** = p<0.001; **** = p<0.0001; ns = p>0.05).

As expected, sdLN CD4^+^ cells from 1x mice were responsive and proliferated *in vitro* to parasite SSAP antigen (Fig 6A, left). The addition of dermal CD4^+^ T cells from 4x infected IL-10^-/-^ mice did not significantly affect the ability of 1x sdLN CD4^+^ cells to proliferate (Fig 6B, p>0.05). However, addition of IL-10 competent dermal CD4^+^ T cells from 4x WT mice significantly inhibited proliferation of 1x sdLN CD4^+^ cells (Fig 6B, p<0.05). Moreover, the difference between adding dermal CD4^+^ T cells from 4x WT and 4x IL-10^-/-^ mice was statistically significant (Fig 6B, p<0.001).

Furthermore, IL-10^GFP+^ dermal CD4^+^ T cells sorted from the DEC of 4x infected mice significantly inhibited proliferation of 1x sdLN CD4^+^ cells in response to parasite SSAP antigen (Fig 6C and 6D, p<0.05), whereas GFP^Neg^ dermal CD4^+^ T cells from the same 4x mice did not significantly alter the proliferation of 1x sdLN CD4^+^ cells (Fig 6C and 6D, p>0.05). Combined, these data demonstrate that IL-10 production from dermal CD4^+^ T cells is responsible for regulating the responsiveness of sdLN CD4^+^ T cells.

## Discussion

The role of IL-10 in maintaining immune homeostasis and resolving inflammation is critical for host survival and function [16,37–39], a phenomenon that has been well studied in the pathogenesis of chronic schistosomiasis [28–31] but is not understood, or documented, at the early stage of percutaneous schistosome infection. Here, we show that repeated exposures of the skin to infective *S*. *mansoni* cercariae results in an early increase in IL-10 production by CD4^+^ T cells at the skin site of infection. The production of IL-10 by these cells occurs prior to the late stage production of IL-10 conventionally associated with chronic schistosome infection after the onset of egg deposition [30,40]. Single infection with *S*. *mansoni* cercariae leads to transient IL-10 production in the skin, which returns to naïve levels after 14 days [41], whilst repeated infection leads to sustained production of IL-10 between days 1–8 after exposure [18]. In our model, early IL-10 production by dermal CD4^+^ T cells was important in limiting the extent of inflammation at the site of infection by reducing skin thickening and neutrophil recruitment. Moreover, dermal CD4^+^ T cells were able to limit local proliferation of CD4^+^ T cells in the dermis and suppress the proliferation of normally responsive CD4^+^ T cells from proximal sdLN of singly infected animals.

In the current study, CD4^+^ T cells were the main producers of IL-10 after repeated exposure to *S*. *mansoni* cercariae supporting the findings of others, who report that CD4^+^ T cells are important contributors to the production of IL-10 in a range of disease settings [16,42–46], as well as being an important target of IL-10 [47]. In the mesenteric lymph nodes and the spleen during chronic *S*. *mansoni* infection, CD25^+^CD4^+^ and CD25^-^CD4^+^ T cells were the main producers of IL-10 [48], and it is well known that IL-10 is important in limiting increased granuloma formation around eggs deposited in the liver, leading to decreased host survival [31,49]. However, here we demonstrate that production of IL-10 by dermal CD4^+^ T cells occurs very early during the initial stages of *S*. *mansoni* infection, well in advance of egg deposition, which is a hallmark of chronic/long term infection. We show that early IL-10 was instrumental in limiting inflammation after primary but particularly following repeated exposures to infectious cercariae. Local proliferation of CD4^+^ T cells, plus the recruitment of neutrophils were regulated by this early production of IL-10 in the skin. This suggests that during natural infection, where repeated exposures to infective cercariae are likely to be common for residents of schistosome-endemic areas [32], IL-10 could play an important role in limiting leukocyte mediated tissue damage associated with migration of larvae through the skin. Indeed, whole blood cultures of infected individuals in endemic regions produced enhanced levels of IL-10 in response to cercarial E/S antigens [26], which supports the hypothesis that this cytokine is triggered by the early stage of the parasite.

Multiple subsets of T helper cells can make IL-10 [16,42–46], including T helper (Th) 2, thymic Nrp1^+^ Helios^+^ T_reg_ (tT_reg_), peripheral FoxP3^+^ Helios^-^ T_reg_ (pT_reg_), and Tr1 cells [27,50,51], and previous studies in the skin have highlighted a role of regulatory T cells in the prevention of excessive inflammation during exposure to protozoan parasites [15–17,52]. However in our studies, the proportions of both tT_reg_ and pT_reg_ decreased in 4x infected mice, and as they were a scarce source of IL-10, there was limited evidence for these regulatory CD4^+^ T cell subtypes being responsible for early IL-10 in the skin of mice repeatedly exposed to larvae of the *Schistosoma* helminth. Although CD223^+^ CD49b^+^ Tr1 cells, which are thought to exert their function through the production of IL-10 [14,50], were slightly increased as a proportion of all CD4^+^ T cells in 4x mice, and as a proportion and number of IL-10^+^ CD4^+^ T cells in the skin, most of the IL-10^+^ CD4^+^ T cells in the skin expressed none of the markers associated with conventional ‘regulatory’ phenotypes despite being functionally suppressive. Nevertheless, as several of these functionally suppressive IL-10^+^ CD4^+^ T cells co-expressed Nrp1 and CD49b, both of which are up-regulated in activated T cells [53,54], our findings do not rule out the possibility that they arise from conventional regulatory T cells which have lost expression of FoxP3, Helios, or CD223 (LAG3), as reported by others [55,56].

A major difference that distinguishes our work from those cited above [15–17,52] is that they were based upon the study of immune responses to a single pathogen exposure leading to persistent/chronic infection. In contrast, in our study, repeated exposures to cercariae led to regulation being evident early after infection, thus emphasizing the early role of antigen-specific CD4^+^ T cells in mediating immune regulation in sites of initial infection, such as the skin. The functionally suppressive CD4^+^ T cells had a putative Th2 bias, as they secreted IL-4, but not IFN-γ, which is in line with our previous studies showing the production of elevated levels of IL-4, but not Th1 associated cytokines in skin exposed to multiple doses rather than a single dose of cercariae [18]. CD4^+^ cells were confirmed as the source of IL-4 since this cytokine was not detected in T cell deficient skin (i.e. Rag2^-/-^). The role of Th2 cells in limiting skin inflammation has been suggested during disorders such as atopic dermatitis, where innate immune cells are regulated by cytokines from Th2 cells [13,57]. However, recent literature highlights the possibility that CD4^+^ T cells could be tissue resident memory cells (TRM) as identified primarily in pulmonary and gastrointestinal mucosal tissue sites [58,59]. Whilst CD69 and CD103 expressing CD4^+^ cells have also been reported in skin [9,60–62], it is not clear whether they are circulating, or are tissue resident, and we did not observe dermal CD69^+^ CD4^+^ T, or CD103^+^ CD4^+^ T cells in our model. Finally, although CD4^+^ TRM cells can produce IFNγ/IL-17 [58], and IL-4/IL-13 following helminth infection [63], their production of IL-10 has not previously been reported. Consequently, further investigation into the possible definition of IL-10^+^ CD4^+^ population in the dermis is warranted.

Myeloid cells such as F4/80^+^MHC-II^high^ tissue resident macrophages were also a source of IL-10 in the skin after *S*. *mansoni* infection, most likely in response to cercarial E/S products [25] (Sanin & Mountford, *manuscript in preparation*). We showed previously that after repeated infection, M2-like dermal APCs conditioned within the skin infection site, which is rich in IL-4 and IL-10, were associated with decreased CD4^+^ T cell responsiveness in the skin draining lymph node [18]. However, our current findings show that proliferation of dermal CD4^+^ T lymphocytes is not impaired in repeatedly infected skin and that IL-10 production is predominantly from CD4^+^ T cells in the skin rather than myeloid cells. This accords with recent findings that IL-10 is the main driver of CD4^+^ T cell hypo-responsiveness in the lymph node [33]. In the present study, the role of F4/80^+^MHC-II^high^ macrophage derived IL-10 might be important for the initial polarization of dermal CD4^+^ T cells towards their subsequent production of IL-10, as shown in another recent study of helminth infection [64]. Indeed, IL-10 is able to enhance its own expression through the activation of STAT3 [65]. The clusters of MHC-II^+^ cells and CD4^+^ T cells observed in schistosome-infected skin may represent how myeloid cells activate CD4^+^ T cells after *S*. *mansoni* infection, as suggested in other infection models [66,67]. IL-10 production in T cells can be regulated by several cytokines (e.g. IL-4, IL-6, IL-12, and IL-27), as well as multiple transcription factors (i.e. STAT1, STAT3, STAT4, STAT6, GATA3, and c-MAF) [68]. In particular, IL-27 has been reported to drive IL-10 production by CD4^+^ T cells in dermal lesions caused by infection with *Leishmania* parasites [69], although IL-27 was not detected in the culture supernatants of skin biopsies in our schistosome infection model. However, full exploration of these cytokines and transcription factors was beyond the scope of the current study.

In the absence of T lymphocytes (i.e. Rag2^-/-^), very low levels of IL-10 were noted and the dermal immune response was equivalent to that in IL-10^-/-^ mice, supporting our conclusion that myeloid and/or CD4^negative^ lymphoid cells are not a major source of IL-10. Other lymphocytes, which are absent in Rag2^-/-^ skin, such as B cells and CD8^+^ T cells were rare in infected skin (<1000 cells) and did not expand in number, or proportion after repeated infection, nor did they produce IL-10. Therefore, IL-10 derived from dermal CD4^+^ T cells, rather than myeloid cells [18], appears to be responsible for inducing CD4^+^ lymphocyte hypo-responsiveness in the sdLN, as well as conditioning the dermal immune environment.

Although functionally suppressive IL-10^+^ CD4^+^ T cells in the dermis had specificity for schistosome antigen, a population of dermal CD4^+^ T cells produced IL-10 in response to antigens from commensal microbiota. Indeed, bacterial commensals in barrier tissues such as the skin can influence the immune response [12,13,70], and can exacerbate immune pathology by altering the balance between regulatory and effector T cells by triggering IL-17 and IFN-γ, although a role for IL-10 has not been established [36,71,72]. Here we show that CD4^+^ T cells in the skin produce IL-10 in a response that was initially directed against commensal microbiota. We speculate that skin commensals gain access to the dermis as the parasites invade the skin and therefore stimulate resident T cells with specificity for antigens from commensal microbiota as early as day 1 after schistosome infection. These early responding T cells could include Tr1 cells, which are a known source of IL-10 [14,50], especially as their antigen specificity can be to commensal microbiota [52]. The early production of IL-10 triggered by commensals as a result of penetrating *S*. *mansoni* cercariae, therefore could be a strategy adopted by the parasite to limit anti-parasite immune responses from the host. This might be particularly relevant in the context of the enhanced neutrophilia observed here in the absence of IL-10. The use of germ-free animals could provide direct evidence of the role of skin commensals in *S*. *mansoni* infection, as topical antibiotic treatment of skin to eliminate skin microbiota is likely to have an adverse effect on cercarial penetration, as seen with the use of soap [73]. However, the impact of commensal microorganisms on the dermal immune response to *Schistosoma* infection in humans in the face of concurrent antibiotic treatment requires further investigation.

In summary, we demonstrate that repeated exposures of the skin to infective *S*. *mansoni* cercariae leads to an early increase in IL-10 production by *S*. *mansoni* specific CD4^+^ T cells in the dermis, with a putative Th2 bias, at the site of infection. This is accompanied by a previously unreported bystander response to commensal microbiota that gain access to the dermis as cercariae penetrate the skin. IL-10 production by these dermal CD4^+^ T cells is important in limiting the extent of skin inflammation, leukocyte proliferation and recruitment. Critically, these dermal CD4^+^ T cells are able to suppress the proliferation of CD4^+^ T cells from the draining lymph nodes which could explain the development of hypo-responsiveness should they migrate *in vivo* to proximal lymph nodes. Collectively our findings demonstrate the importance of early IL-10 production by functionally suppressive CD4^+^ T cells in the skin in response to *S*. *mansoni* cercariae and highlights a possible role of these cells in maintaining host fitness in populations that inhabit areas endemic for schistosomiasis, and other helminth larvae that penetrate via the skin [74,75]. Finally, our data show that functionally suppressive CD4^+^ T helper cells, that are not conventional regulatory CD4^+^ T cells, are important as modulatory cells in the skin after repeated exposure to pathogens. We suggest that the role of IL-10 in controlling early immune responses to schistosomes, may act as a prelude to the subsequent development of IL-10 mediated immune regulation conventionally associated with chronic helminth infections.

## Materials and Methods

### Ethics statement

Female mice aged between 6–10 weeks were used for all experiments carried out in accordance with the UK Animal’s Scientific Procedures Act 1986 and with approval of the University of York Ethics Committee (PPL #60/4340).

### Animals

Wild type C57BL/6 (WT) and IL-10 deficient (IL-10^-/-^) mice [76], as well as transgenic mice lacking RAG2 (Rag2^-/-^), or IL-10 reporter knockin (*tiger*) animals (IL-10^+/GFP^) [34] were bred and housed at the University of York.

### Infection protocol and parasites

Mice were percutaneously exposed via each pinna to four doses (4x) of 150 *S*. *mansoni* cercariae at weekly intervals from day 0 to day 21 as previously described (repeated infection) [18,77]. Age and sex-matched cohorts were exposed in parallel to a single dose (1x) of 150 cercariae on day 21. Using this infection protocol via the pinna, a 50% penetration rate is observed [77] amounting to ~75 cercariae per pinna at each time-point. Inflammation of pinnae was measured using a dial gauge micrometer (Mitutoyo, Japan). In most experiments, pinnae were harvested 4 days after the last infection, or in selected experiments obtained on day one. Auricular lymph nodes draining the skin site of infection (sdLN) were also harvested in specific experiments on day 4 post-final infection.

### Dermal exudate cells

Pinnae from naïve, and infected mice (on day 1 or 4 post-final exposure to cercariae) were removed, briefly sterilized with ethanol, air-dried and split along the central cartilage into two halves to obtain the population of dermal exudate cells (DEC) as described previously [18,78]. Split pinnae were floated overnight at 37°C 5% CO_2_ in the absence of added antigen on RPMI-1640 media (Gibco, Paisley, UK) containing 10% heat inactivated FCS (Biosera, Uckfield, UK), 2mM L-Glutamine solution, 1% Pen/Strep (both Gibco), and 50μM 2-mercaptoethanol (Sigma-Aldrich, Gillingham, UK) (complete RPMI) in non-adherent 24 well tissue culture plates (Greiner Labortechnik, Frickenhausen, Germany). Floating pinnae tissues were removed and the remaining culture supernatants containing DEC spun at 1000g for 7min at 4°C before being re-suspended in complete RPMI and live cells enumerated using a hemocytometer. Cell-free culture supernatants were recovered and stored at -20°C before being analyzed by cytokine-specific ELISA.

### *In vitro* antigen stimulation of DEC

In order to stimulate DEC for an antigen-specific recall response, soluble schistosomula antigen preparation (SSAP) was prepared from *in vitro* cultured mechanically-transformed larvae as described previously [79]. Skin commensal antigen (SC) was prepared by culturing skin swabs taken from female WT C57BL/6 mice overnight in liquid broth medium at 37°C. Recovered microbes were washed in sterile PBS, sonicated at full power in PBS for 5 min (30s on / 30s off), and inactivated for 0.5h under UV light. DEC were then cultured at 5x10^5^ cells/ml in complete RPMI in the presence, or absence, of 50 μg/ml SSAP, or 5 μg/ml SC, for 96 hours at 37°C. Proliferation was measured after labelling DEC with 3 μM CFDA-SE (Invitrogen, Paisley, UK) [18]. DEC were subsequently labelled with specific monoclonal antibodies (mAb) detailed below and proliferation determined by flow cytometry according to the decrease in the fluorescence of CFDA-SE.

### Flow cytometry

DEC were incubated with 1 μg anti-CD16/32 mAb (eBioscience, Hatfield, UK) in goat-serum (Sigma-Aldrich) to block non-specific uptake of antibodies and then subsequently labelled with LIVE/DEAD Fixable Aqua Dead Cell Stain (Life Technologies, Paisley, UK) according to the manufacturer’s instructions, plus the following mAbs conjugated to various fluorescent labels: anti-CD4 (clone RM4-5), anti-CD3 (clone 17A2), anti-MHC-II (IA-IE) (clone M5/114), anti-Nrp1 (clone 3DS304M), anti-CD11b (clone M1/70), anti-F4/80 (clone BM8), anti-Gr1 (clone RB6-8C5), anti-SiglecF (clone eBio440c), anti-CD45 (clone 2D1) (all eBioscience), anti-CD49b (clone R1-2) (BioLegend, London, UK) and anti-CD223 (LAG3) (clone C9B7W) (BD Bioscience, Oxford, UK). For intracellular staining, cells were washed with and fixed with 2% paraformaldehyde for 1 hour at 4°C before being washed and incubated for 1 hour in 1x permeabilization buffer (eBioscience) for anti-FoxP3 (clone NRRF-30) and anti-Helios (clone 22F6) (eBioscience). All flow cytometry was acquired using the Cyan ADP analyser (DakoCytomation, Stockport, UK), or BD LSR Fortessa analyser (BD Biosciences, Oxford, UK). Data was analysed using FlowJo software v7.6.5 (Tree Star, Inc, Ashland, Oregon, USA).

### Detection of intracellular IL-10 in DEC

Detection of IL-10 production by different cell types in DEC was achieved using IL-10^+/GFP^ mice. WT and IL-10^+/GFP^ mice were infected and pinnae harvested as described above. Split pinnae were incubated with complete RPMI for 12 hours prior to the addition of 1x Brefeldin A (eBioscience) following the manufacturer’s instructions. After a further 8 hours, DEC were prepared for flow cytometric analysis, by washing once with PBS.

### Sorting of dermal CD4^+^ T cells and *in vitro* co-culture with sdLN cells

DEC obtained from 4x infected WT, IL-10^+/GFP^ and IL-10^-/-^ mice were labelled with mAb against CD4, CD3, CD45 and MHC-II as described above. Dermal CD45^+^CD3^+^CD4^+^ T cells were recovered following FACS (MoFlo Astrios, Beckman Coulter, London, UK) from WT and IL-10^-/-^ mice. For IL-10^+/GFP^ mice, dermal CD45^+^CD3^+^CD4^+^ IL-10^GFP+^ T cells and CD45^+^CD3^+^CD4^+^ IL-10^GFP-^ T cells were obtained. CFDA-SE labelled single cell suspensions from the sdLN were co-cultured at 2.5x10^5^ cell/ml in complete RPMI with, or without, 2x10^3^ sorted dermal T cells in the presence, or absence, of 50 μg/ml SSAP for 96 hours at 37°C. Antigen-specific proliferation of sdLN CD4^+^ cells was measured by a decrease in levels of CFDA-SE after 72 hours as described above compared to cells stimulated the absence of antigen.

### Cytokine analysis by ELISA

Culture supernatants were collected from overnight skin biopsies, or from sdLN cell cultures after 96 hours, for cytokine analysis as previously described [80]. IL-4, IL-10 and IL-12p40 were quantified by DuoSet ELISA (R&D Systems, Abingdon, UK), whilst IFN-γ was measured using specific capture and detection antibodies (BD Pharmingen, Oxford, UK) [77].

### *In vivo* cell proliferation

Mice received 1 mg BrdU (Sigma-Aldrich) i.p. daily for the final 4 days before harvest of pinnae in order to determine *in vivo* cell proliferation. DEC were then recovered, and blocked with 1 μg anti-CD16/32 mAb in goat-serum. Subsequently, DEC were labelled for surface expression of CD3, CD4, MHC-II, F4/80 and CD11b (all eBioscience) in PBS supplemented with 1% FCS. Cells were then washed and incubated in 1x Fixation/Permeabilisation buffer (eBioscience) for 1 hour at 4°C before being washed and incubated for 1 hour at 37°C in 100 μg DNase (Sigma-Aldrich). Finally, cells were labelled for 45 minutes at room temperature with anti-BrdU APC mAb, or rat IgG1 APC (eBioscience), in 1x Permeabilisation buffer as per the manufacturer’s protocol.

### Confocal microscopy and immunofluorescence

Freshly recovered pinnae were fixed in PBS/4% paraformaldehyde on ice for 30 min then transferred to PBS/15% sucrose for a further 1h on ice. Fixed pinnae were then embedded in OCT medium (Sakura Finetek, Netherlands), and frozen at -80°C. Cryosections (6μm) obtained from frozen pinnae were simultaneously blocked and permeabilised in PBS supplemented with 5% goat serum (Sigma-Aldrich) and 0.05% saponin (Sigma-Aldrich) for 30min at room temperature. Sections were incubated in PBS/5% goat serum/0.05% saponin for 1h at room temperature with mAbs directly conjugated to various fluorescent labels: anti-CD4 (clone RM4-5), anti-MHC-II (IA-IE) (clone M5/114) and anti-CD11b (clone M1/70), or suitable isotype controls (all eBioscience) and then washed in PBS/0.05% saponin (3x, 5min each). Finally, sections were counter-stained with 2μg/ml DAPI (Life Technologies) for 5min and rinsed with distilled water. Slides were mounted in Prolong Gold AntiFade reagent (Life Technologies) prior to analysis using a Zeiss 710 inverse confocal microscope (Carl Zeiss, Cambridge, UK). All images were analysed using identical acquisition settings in Zeiss ZEN software. Image handling (including contrast adjustment) was performed on ImageJ (National Institute of Health).

### Statistics

Analysis of variance (ANOVA) and then multiple comparisons tests (Bonferroni’s, Tukey’s, Sidak’s and Dunnett’s) were performed to establish significant differences between the groups (* = p<0.05, ** = p<0.01; *** = p<0.001, **** = p<0.0001) using the software package GraphPad Prism (GraphPad Software, Inc., La Jolla, California, USA). Similarly, unpaired two-tailed T tests were performed in selected experiments to compare experimental groups. Error bars represent the standard error of the mean (SEM), based on technical replicates for *in vitro* experiments, or biological replicates for *in vivo* experiments.

### Accession numbers

**Table:** 

Protein	GenBank Accession number
CD11b	ABK96806
CD223	NP_032505
CD3	NP_031674
CD4	AAC36010
CD49b	NP_032422
F4/80	NP_034260
FoxP3	NP_001186277
Gr1	NP_034474
Helios	NP_035900
IFN-γ	ACR22511
IL-10	EDL39722
IL-4	CAA28731
Nrp1	AAH51447
SiglecF	EDL22650

## Supporting Information

S1 Fig Gating strategy for Dermal Exudate Cells. (**A**) Representative flow cytometry dot plots of live DEC from 1x and 4x infected WT mice, based on F4/80 and MHC-II expression, showing F4/80^-^MHC-II^-^ leukocytes (R1), F4/80^+^MHC-II^-^ (R2) eosinophils, F4/80^+^MHC-II^mid^ (R3) blood derived macrophages, F4/80^-^MHC-II^high^ (R4) blood-derived DCs and F4/80^+^MHC-II^high^ (R4A) tissue resident macrophages. (**B**) Division of R1 gate based on CD4 and CD11b expression into CD11b^high^CD4^-^Gr1^+^ neutrophils (upper panel), and CD11b^-^CD4^+^CD3^+^ T cells (lower panel). Relevant isotype controls for all antibodies are presented next to the corresponding flow plot.
(TIFF)

S2 Fig Changes in DEC populations in the absence of IL-10. (**A**) Representative flow cytometry dot plots of SiglecF^+^F4/80^+^MHC-II^-^ eosinophils in DEC recovered from infected 1x or 4x WT and IL-10^-/-^ mice. Proportions of (**B**) R3 (F4/80^+^MHC-II^mid^) (**C**) R4 (F4/80^-^MHC-II^high^) and (**D**) R4A (F4/80^+^MHC-II^high^) cells of total DEC recovered from 1x, or 4x infected WT and IL-10^-/-^ mice on day 4 post-final exposure. Symbols are values for cells obtained from independent tissue samples; horizontal bars are the means ± SEM; n = 4–8 pinnae per group. Means of selected groups were compared by ANOVA and multiple comparisons tests (Bonferroni’s and Sidak’s) analysis (* = p<0.05; ** = p<0.01; *** = p<0.001; **** = p<0.0001; ns = p>0.05).
(TIFF)

S3 Fig Visualisation of CD4^+^ T cells and antigen presenting cells into 4x infected skin. Confocal images of pinnae cryosections incubated with mAbs specific for CD4 (green), MHC-II (red) and CD11b (yellow), plus DAPI as a nuclear stain (blue), from (**A**) 1x WT, (**B**) 4x WT or (**C**) 4x IL-10^-/-^ skin. (**D**) Isotype controls for each antibody were used as negative controls. Scale bar = 50μm.
(TIFF)

S4 Fig IL-10 production by DEC in naïve skin and on day 1 after exposure to *S*. *mansoni* cercariae. (**A**) Proportion of IL-10^GFP+^ cells in DEC from naive mice with cells defined as R3 (F4/80^+^MHC-II^mid^), R4 (F4/80^-^MHC-II^high^), R4A (F4/80^-^MHC-II^high^) and CD4^+^ T cells (n = 4 pinnae) and (**B**) from 1x infected mice (n = 6 pinnae) on day 1 day after exposure to cercariae (n = 6 pinnae). ANOVA and Sidak’s multiple comparisons test were performed to find statistically significant differences between the means of selected groups (*** = p<0.001; **** = p<0.0001; ns = p>0.05).
(TIFF)

S5 Fig Dermal CD4^+^ T cells from naïve skin proliferate and produce IL-10 after *in vitro* stimulation with commensal microbial antigen. (**A**) Number of IL-10^GFP+^ CD4^+^ T cells in DEC from naive mice and on day 1 day after exposure to cercariae (n = 4–5 pinnae). A T-test was performed to compare the means of selected groups (* = p<0.05). (**B**) Flow cytometry density plots of CFDA-SE^dim^ CD3^+^CD4^+^ dermal T cells from naïve, or infected mice recovered on day 1 after infection; DEC were obtained from skin biopsies and stimulated *in vitro* for 96h in the presence, or absence of parasite antigen (SSAP) or skin commensal antigen (SC). Production of (**C**) IL-10, (**D**) IL-4 and (**E**) IFN-γ in culture supernatants of skin biopsies from naïve mice cultured in the presence, or absence of SSAP or SC antigen; bars are means + SEM, n = 3.
(TIFF)

S6 Fig Non-CD4^+^ lymphocytes in DEC from skin exposed to *S*. *mansoni* cercariae. Absolute numbers of live (**A**) CD3^+^CD8^+^ and (**B**) CD3^-^B220^+^ lymphocytes in DEC recovered from mice 4 days after a single (1x) or repeated (4x) infection with *S*. *mansoni* cercariae. Symbols are values for cells obtained from independent tissue samples; horizontal bars are the means ± SEM; n = 12 pinnae per group. The means of selected groups were compared via unpaired T test (* = p<0.05; ns = p>0.05).
(TIFF)
