# Supplementary figures and images for: Helminth Infection and Commensal Microbiota Drive Early IL-10 Production in the Skin by CD4+ T Cells That Are Functionally Suppressive
Source: PLoS Pathog. 2015 May 14;11(5):e1004841. doi: 10.1371/journal.ppat.1004841 (PMC4431738; doi:10.1371/journal.ppat.1004841)

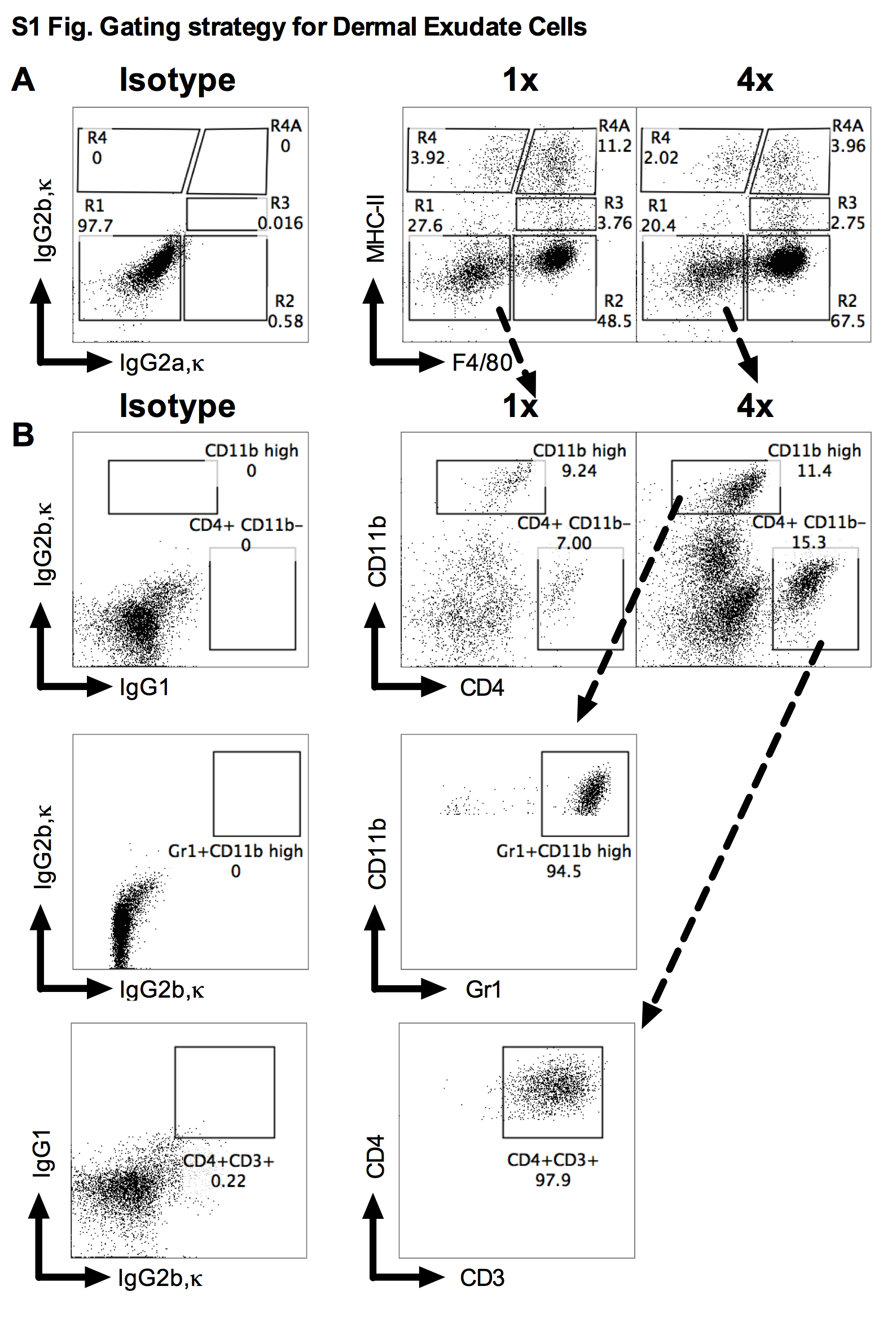

Supplement: S1 Fig — (A) Representative flow cytometry dot plots of live DEC from 1x and 4x infected WT mice, based on F4/80 and MHC-II expression, showing F4/80-MHC-II- leukocytes (R1), F4/80+MHC-II- (R2) eosinophils, F4/80+MHC-IImid (R3) blood derived macrophages, F4/80-MHC-IIhigh (R4) blood-derived DCs and F4/80+MHC-IIhigh (R4A) tissue resident macrophages. (B) Division of R1 gate based on CD4 and CD11b expression into CD11bhighCD4-Gr1+ neutrophils (upper panel), and CD11b-CD4+CD3+ T cells (lower panel). Relevant isotype controls for all antibodies are presented next to the corresponding flow plot. (TIFF) [file ppat.1004841.s001.tiff]

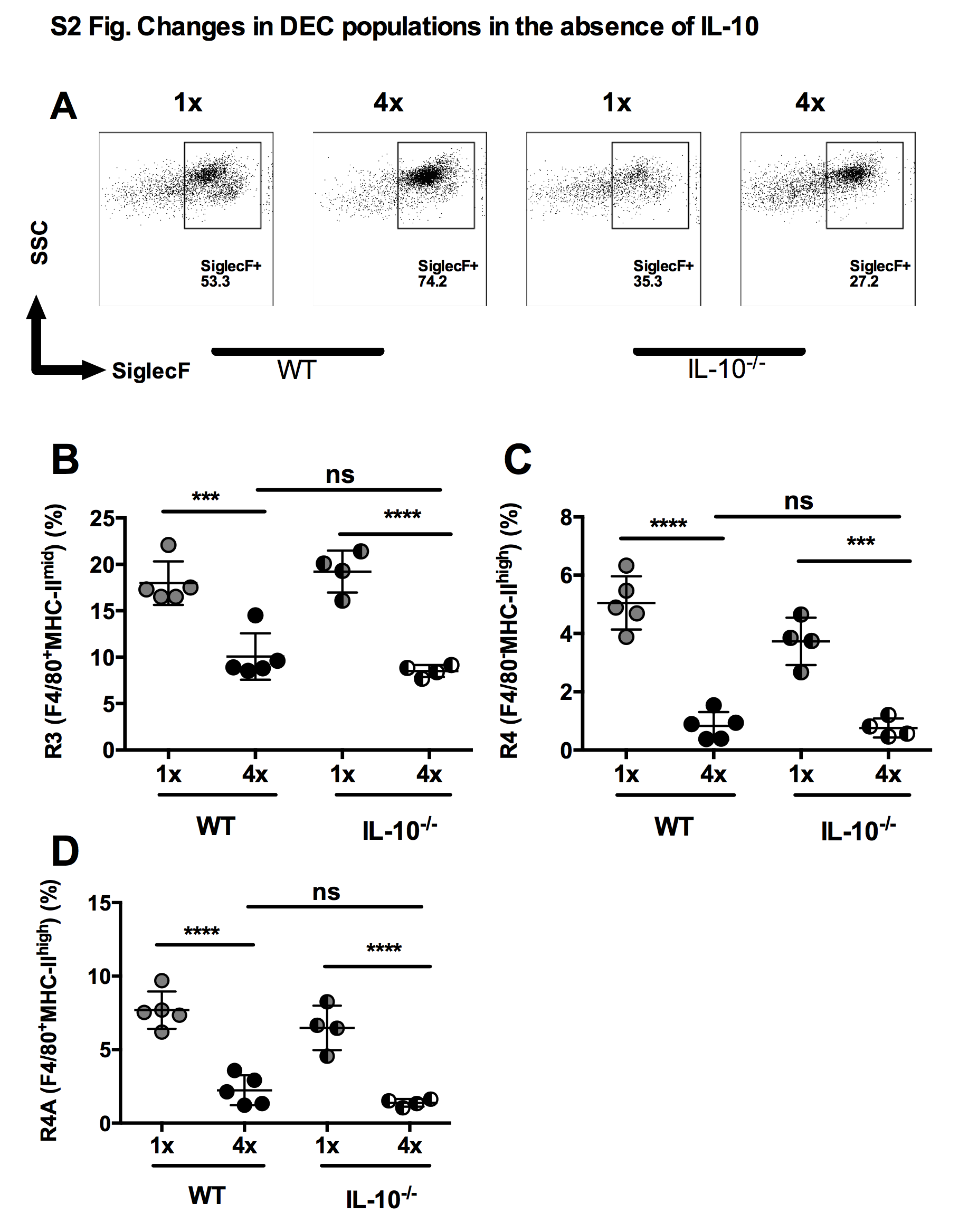

Supplement: S2 Fig — (A) Representative flow cytometry dot plots of SiglecF+F4/80+MHC-II- eosinophils in DEC recovered from infected 1x or 4x WT and IL-10-/- mice. Proportions of (B) R3 (F4/80+MHC-IImid) (C) R4 (F4/80-MHC-IIhigh) and (D) R4A (F4/80+MHC-IIhigh) cells of total DEC recovered from 1x, or 4x infected WT and IL-10-/- mice on day 4 post-final exposure. Symbols are values for cells obtained from independent tissue samples; horizontal bars are the means ± SEM; n = 4–8 pinnae per group. Means of selected groups were compared by ANOVA and multiple comparisons tests (Bonferroni’s and Sidak’s) analysis (* = p<0.05; ** = p<0.01; *** = p<0.001; **** = p<0.0001; ns = p>0.05). (TIFF) [file ppat.1004841.s002.tiff]

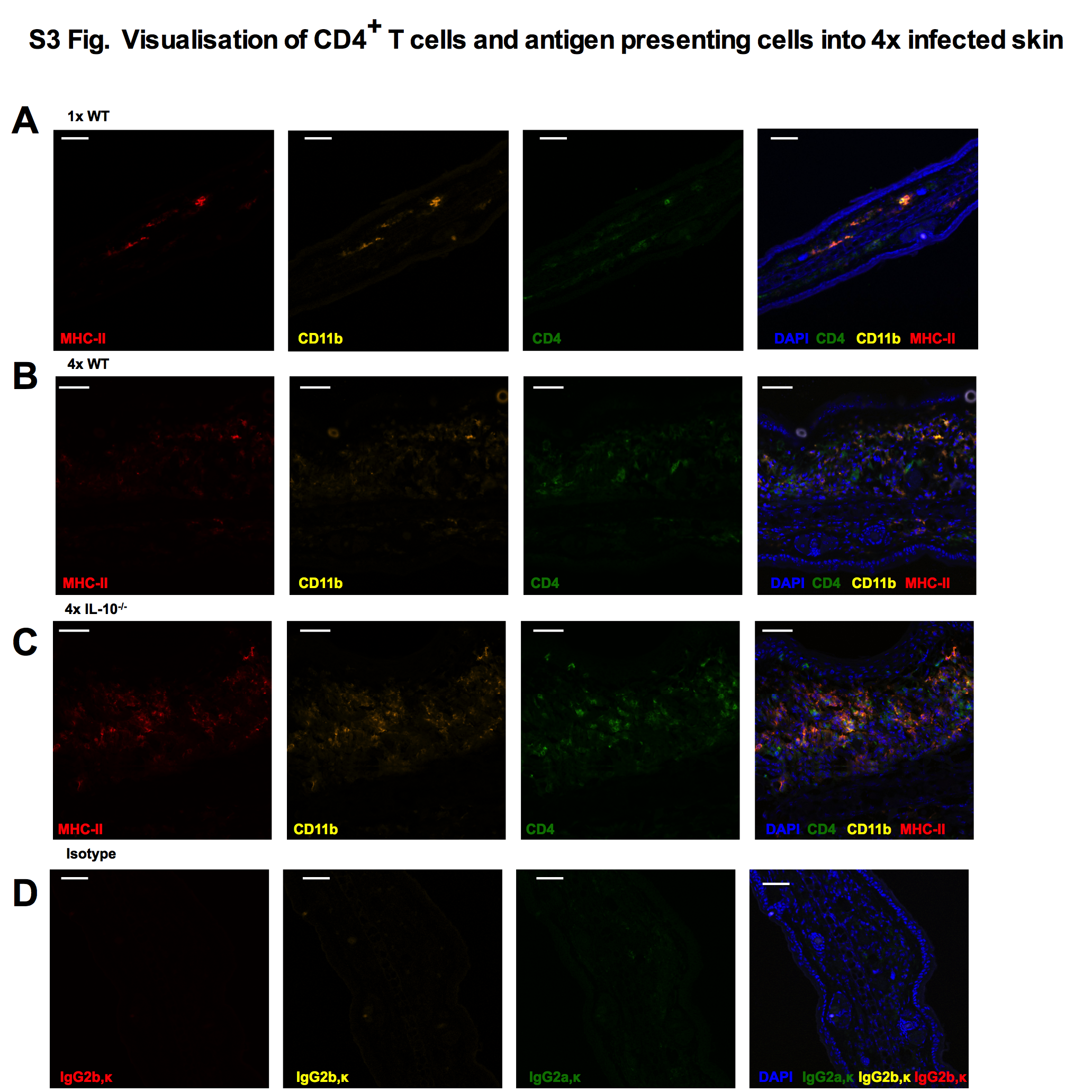

Supplement: S3 Fig — Confocal images of pinnae cryosections incubated with mAbs specific for CD4 (green), MHC-II (red) and CD11b (yellow), plus DAPI as a nuclear stain (blue), from (A) 1x WT, (B) 4x WT or (C) 4x IL-10-/- skin. (D) Isotype controls for each antibody were used as negative controls. Scale bar = 50μm. (TIFF) [file ppat.1004841.s003.tiff]

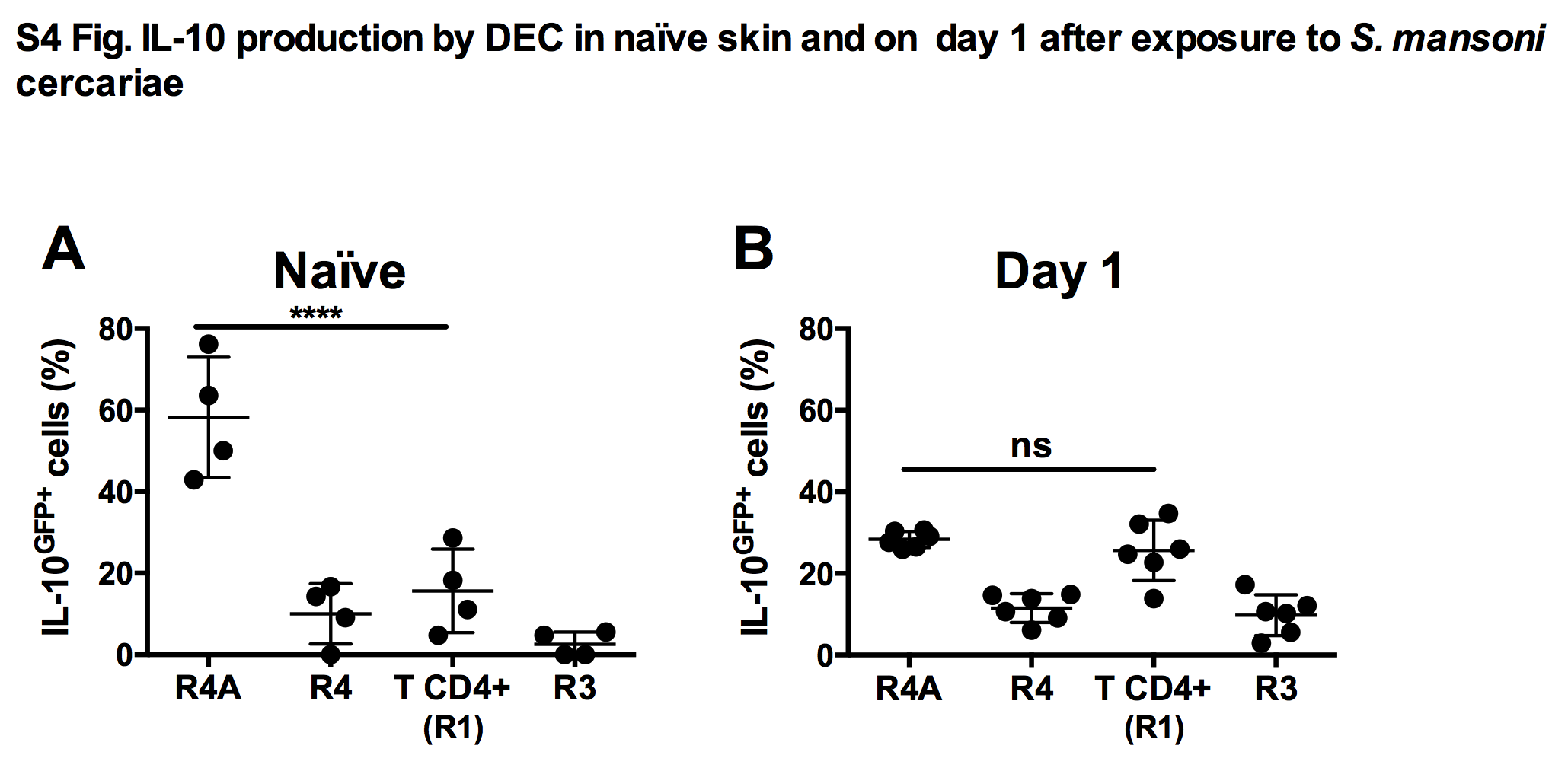

Supplement: S4 Fig — (A) Proportion of IL-10GFP+ cells in DEC from naive mice with cells defined as R3 (F4/80+MHC-IImid), R4 (F4/80-MHC-IIhigh), R4A (F4/80-MHC-IIhigh) and CD4+ T cells (n = 4 pinnae) and (B) from 1x infected mice (n = 6 pinnae) on day 1 day after exposure to cercariae (n = 6 pinnae). ANOVA and Sidak’s multiple comparisons test were performed to find statistically significant differences between the means of selected groups (*** = p<0.001; **** = p<0.0001; ns = p>0.05). (TIFF) [file ppat.1004841.s004.tiff]

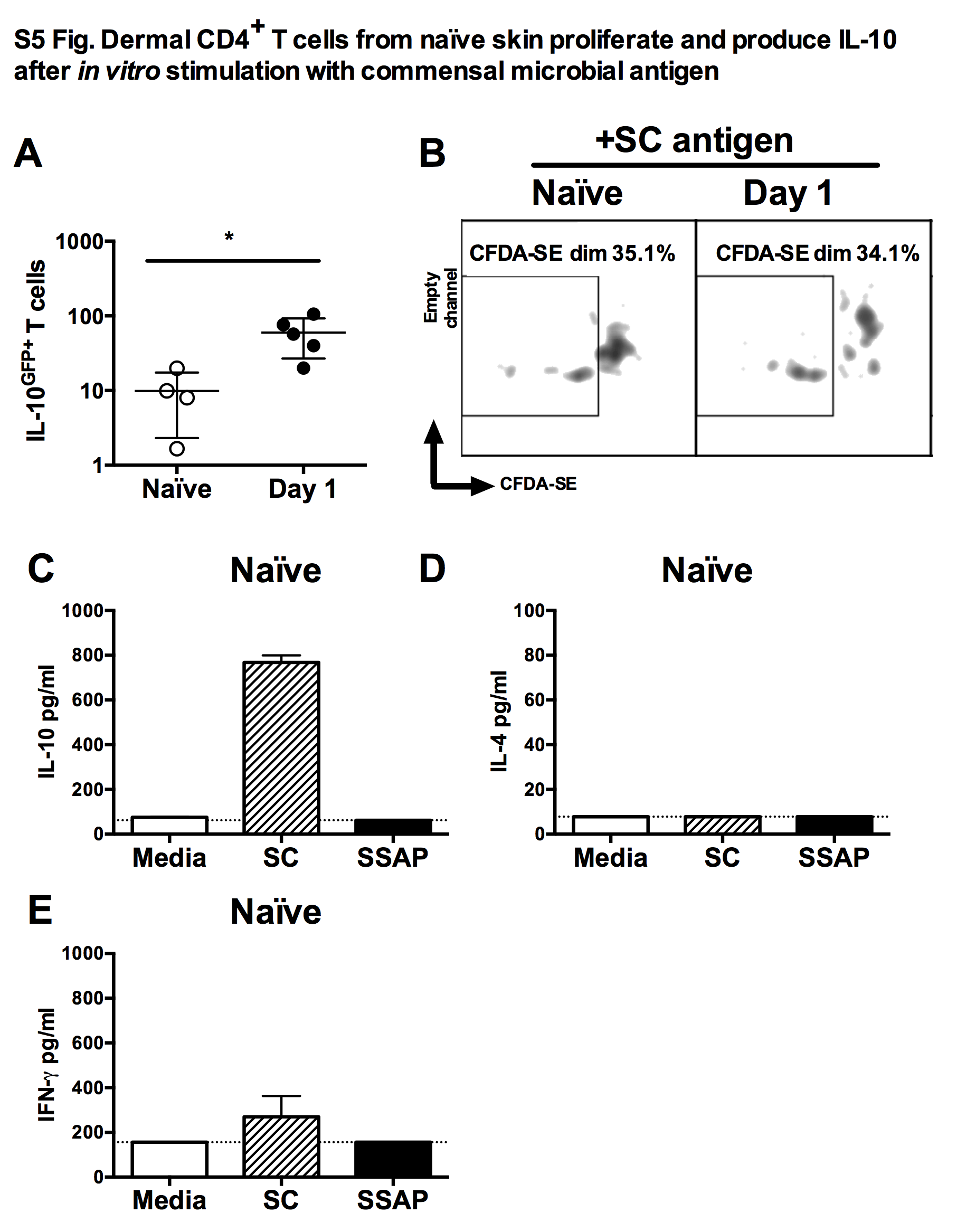

Supplement: S5 Fig — (A) Number of IL-10GFP+ CD4+ T cells in DEC from naive mice and on day 1 day after exposure to cercariae (n = 4–5 pinnae). A T-test was performed to compare the means of selected groups (* = p<0.05). (B) Flow cytometry density plots of CFDA-SEdim CD3+CD4+ dermal T cells from naïve, or infected mice recovered on day 1 after infection; DEC were obtained from skin biopsies and stimulated in vitro for 96h in the presence, or absence of parasite antigen (SSAP) or skin commensal antigen (SC). Production of (C) IL-10, (D) IL-4 and (E) IFN-γ in culture supernatants of skin biopsies from naïve mice cultured in the presence, or absence of SSAP or SC antigen; bars are means + SEM, n = 3. (TIFF) [file ppat.1004841.s005.tiff]

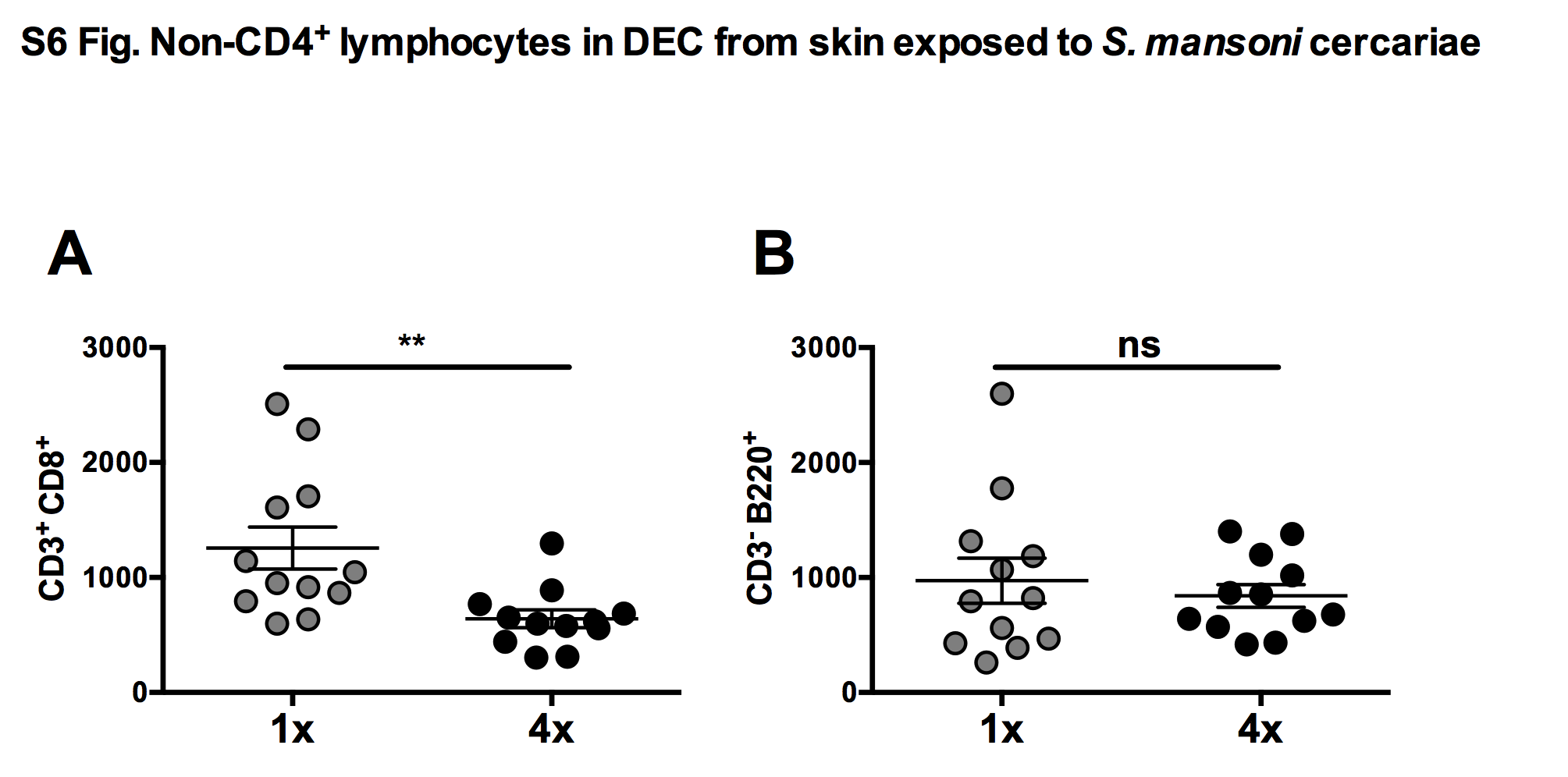

Supplement: S6 Fig — Absolute numbers of live (A) CD3+CD8+ and (B) CD3-B220+ lymphocytes in DEC recovered from mice 4 days after a single (1x) or repeated (4x) infection with S. mansoni cercariae. Symbols are values for cells obtained from independent tissue samples; horizontal bars are the means ± SEM; n = 12 pinnae per group. The means of selected groups were compared via unpaired T test (* = p<0.05; ns = p>0.05). (TIFF) [file ppat.1004841.s006.tiff]
